# Prediction and Control of Thermal Transport at Defective State Gr/*h*-BN Heterojunction Interfaces

**DOI:** 10.3390/nano13091462

**Published:** 2023-04-25

**Authors:** Mingjian Zhou, Liqing Liu, Jiahao Liu, Zihang Mei

**Affiliations:** School of Mechanical Engineering, Chaohu University, Chaohu 238000, China

**Keywords:** graphene heterojunctions, molecular dynamics, interfacial thermal conductivity, neural network prediction

## Abstract

The control of interfacial thermal conductivity is the key to two−dimensional heterojunction in semiconductor devices. In this paper, by using non−equilibrium molecular dynamics (NEMD) simulations, we analyze the regulation of interfacial thermal energy transport in graphene (Gr)/hexagonal boron nitride (*h*-BN) heterojunctions and reveal the variation mechanism of interfacial thermal energy transport. The calculated results show that 2.16% atomic doping can effectively improve interfacial heat transport by more than 15.6%, which is attributed to the enhanced phonon coupling in the mid−frequency region (15–25 THz). The single vacancy in both N and B atoms can significantly reduce the interfacial thermal conductivity (ITC), and the ITC decreases linearly with the increase in vacancy defect concentration, mainly due to the single vacancy defects leading to an increased phonon participation rate (PPR) below 0.4 in the low-frequency region (0–13 THz), which shows the phonon the localization feature, which hinders the interfacial heat transport. Finally, a BP neural network algorithm is constructed using machine learning to achieve fast prediction of the ITC of Gr/*h*-BN two-dimensional heterogeneous structures, and the results show that the prediction error of the model is less than 2%, and the method will provide guidance and reference for the design and optimization of the ITC of more complex defect-state heterogeneous structures.

## 1. Introduction

Following the successful preparation of graphene, it has received widespread attention in scientific research and industrial fields for its excellent mechanical, optical, electrical, and thermal properties [1,2,3,4,5], in addition to driving the rapid development of other members of the two-dimensional materials family. In recent years, scientists have heterogeneously integrated nanomaterials with small differences in lattice matching to construct specific functions or to achieve synergistic enhancement of comprehensive material properties [6,7]. For example, the heterogeneous integration of boron nitride and graphene is based on their identical molecular structures with small lattice differences [8]. However, the formation of heterogeneous structures of different materials creates interfacial thermal resistance, which can greatly affect the thermal transport of heterogeneous materials, a fact also demonstrated in previous reports [9,10,11]. Therefore, the interfacial thermal management of heterogeneous materials is an area worthy of further study. Based on its dielectric properties and high thermal conductivity, nanoscale boron nitride is widely used as a substrate material for high-performance devices, effectively enhancing the strength and thermal conductivity of the devices [12,13,14,15]. It is well known that defects such as doping and vacancies are inevitable in the preparation of Gr/*h*-BN heterojunctions, and it is worthwhile to investigate how to regulate the interfacial thermal conductivity of heterojunctions and understand their thermal transport mechanisms through defect engineering.

The influence of defect states on ITCs with heterogeneous structures is rich and complex and it is difficult to comprehensively describe the influencing factors of the properties. Moreover, molecular dynamics simulations cannot achieve large-scale optimization to calculate the physical properties through traditional theoretical calculations [16,17]. In recent years, the use of deep learning to design the properties of heterogeneous composites has received much attention [18,19,20], for example, Li et al. [21] used deep learning algorithms to establish the mapping between the topological properties of artificial phonon crystals and acoustic wave propagation properties. Deep learning-based neural networks can establish the intrinsic connection between macroscopic mechanical properties and microstructure, in addition, to accurately and efficiently predicting the mechanical properties of multicomponent composites. Wan et al. [22] predicted the thermal conductivity under different types of graphene void distribution using convolutional neural networks and predicted the graphene pore structure with the lowest thermal conductivity by the model. Inspired by their work [23,24,25], we focus on using machine learning to construct BP neural network algorithms to predict the interfacial thermal conductivity of graphene/h-boron nitride 2D heterostructures and improve the interfacial heat transport at GR/*h*-BN heterogeneous structures interfaces.

In this paper, a non-equilibrium molecular dynamics (NEMD) approach is used to investigate how to modulate the interfacial thermal energy transport at the GR/*h*-BN heterojunction interface. The ITC for doping and vacancy control of the Gr/*h*-BN heterogeneous structures is proposed, and the change mechanism is explained in detail using PDOS and PPR. The results of the simulation calculations are used as a data set to construct a BP neural network machine learning model for fast prediction of the ITC of graphene/*h*-BN heterojunction and compare it with the ITC calculated by molecular dynamics simulations, and then evaluate the accuracy of the BP neural network model.

## 2. Model and Computational Methods

**Figure 1:**
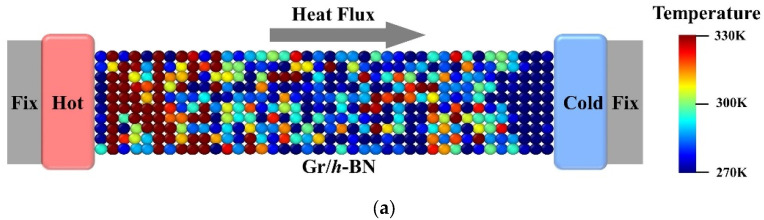

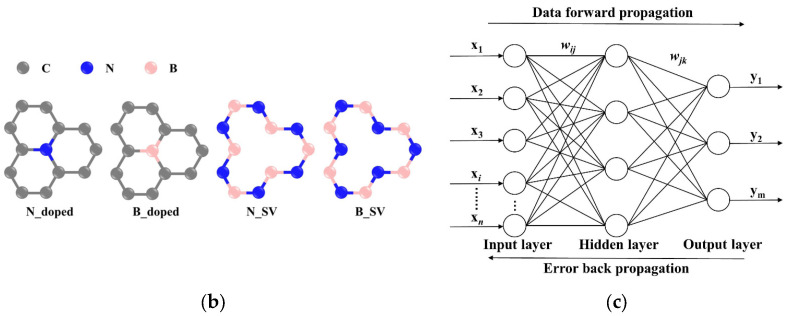
Calculation of interfacial thermal conductivity ITC using NEMD method. (**a**) NEMD temperature distribution map. (**b**) Atomic doping and vacancy structure diagram. (**c**) Schematic diagram of BP neural network.

We constructed the Gr/*h*-BN heterogeneous interface model directly in the massive atomic/molecular massively parallel simulator package (LAMMPS) and performed the subsequent simulations using the NEMD method [26,27]. In the simulations, Gr/*h*-BN has a zigzag shape along the Y-axis and an armchair shape along the X-axis, and all the simulated systems have dimensions of l = 200 Å and w = 50 Å in the X- and Y-axes, respectively. According to the basic principles of molecular dynamics, a suitable potential function needs to be determined to describe the simulated systems to ensure the reliability of the computational results. Based on the experience of previous studies, we use the optimized Tersoff potential [28] to characterize the covalent interactions between C, N, and B atoms. It is important to note that the optimized Tersoff potential predicts the phonon dispersion curves of graphite in close agreement with experimental measurements [29], and these potential parameters set for C, N and B interactions, developed by Kınacı et al. [30], which are especially improved to examine the thermal properties of nanostructures and they are given in Table 1. To eliminate the size effects, a periodic boundary condition is used along the y-direction of the Gr/*h*-BN, and the z and x directions are kept free. In order to avoid the influence between adjacent layers, a vacuum region with a 15 Å space is placed above the surface of hybrid Gr/*h*-BN, and a Langevin thermostat was used to control the temperatures of the cold and hot baths in the X direction as shown in Figure 1a. The N and B atom doping as well as the single vacancy structure diagrams are shown in Figure 1b.

The Verlet algorithm was used to integrate the equations of motion in time steps of 0.0005 ps. The temperature of the system is controlled by a Nose-Hoover heat bath. After the modeling is completed, the velocity of motion of the atoms is initialized given the corresponding temperatures and the model is energy minimized using the most rapid descent method (CG). The whole system is relaxed for 1 ns using isobaric isothermal tandem (NPT) and isobaric isothermal tandem (NVT), respectively, to ensure the steady-state structure. Finally, the equilibrium system is switched to the NVE system to sample the local temperature and cumulative energy exchange. The interfacial thermal conductivity *K* is obtained from Equation (1).

(1) $$K=\frac{J}{∆T}$$

where the heat flow and temperature jumps are *J* and Δ𝑇, respectively.

The transport of phonons between two interface materials can be determined by the overlap of the phonon density of states between them. PDOS can be determined [31,32] using the velocity autocorrelation function of the Fourier transform of all atoms and is calculated by Equation (2).

(2) $$\mathrm{PDOS}\left(\omega \right)=\int_{-\infty }^{+\infty }\langle \frac{1}{N}\sum_{i=1}^{N}\vec{v}i\left(t0\right)-\vec{v}i\left(t0+t\right)\rangle e^{-2\pi i\omega t}dt$$

where $v\left(t\right)$ is the velocity of the *i*th atom at time *t*, *N* is the total number of atoms and the ω represents the phonon frequency, and PDOS (ω) equal to 0 means that no phonons are involved at the frequency.

To quantify the degree of matching in PDOS, the overlap factor *S* is used to calculate the matching degree of PDOS, which is calculated by Equation (3).

(3) $$S=\int_{0}^{\infty }\min\left\{P_{Gr}\left(\omega \right),P_{h-BN}\left(\omega \right)\right\}d\omega$$

where *P_Gr_* (ω) and *P_h-BN_* (ω) denote the PDOS of graphene and *h*-BN at frequency ω, respectively.

It is important to note that we rely on PDOS alone to explain the interfacial heat transport mechanism too singularly. Therefore, we further capture the phonon activity and its effect on ITC in heterogeneous structure systems with different vacancy defect concentrations. The mechanism of ITC variation at different defect concentrations is revealed by measuring the PPR. MD simulations can be used to calculate the PPR at arbitrary temperatures, implicitly for all non-tuning and scattering orders, and in our work, a PPR > 0.4 is used to guarantee the non-localization of most of the phonon modules, and the PPR is calculated by Equation (4).

(4) $$\mathrm{PPR}(\omega )=\frac{1}{N}\frac{{\left(\sum_{i}{\mathrm{PDOS}}_{i}{\left(\omega \right)}^{2}\right)}^{2}}{\sum_{i}{\mathrm{PDOS}}_{i}{\left(\omega \right)}^{4}}$$

The BP neural network is a more commonly used artificial neural network, and its topology structure is shown in Figure 1c. The BP neural network is a multilayer pre-feedback structure, with no information interaction between neuron nodes in the same layer and information transfer between different layers according to the connection weights. The error backpropagation and multilayer design of the BP neural network enable it to reflect the mapping relationship between input and output more accurately in order to accomplish complex tasks. The transfer function of the BP neural network is divided into linear and nonlinear, the nonlinear transfer function generally uses the Sigmoid function, which can be divided into Log-Sigmoid and Tan-Sigmoid functions according to the different ranges of output values. The BP neural network is trained by error backpropagation, the error is calculated by the output layer, and the corrected weights are passed forward layer by layer through the implicit layer, and the BP neural network stops training after the final error becomes smaller to meet the system requirements through repeated iterations.

(5) $$\left\{\begin{array}{c} f(x)=\frac{1}{1+e^{-x}},f(x)\in \left[0,1\right]\\ f(x)=\frac{1-e^{-x}}{1+e^{-x}},f(x)\in \left[-1,1\right] \end{array}\right.$$

The BP neural network with a single input layer, three hidden layers, and a single output layer will be built in this study, where the prediction model is set with five input variables and one output variable, i.e., Gr/*h*−BN heterojunction ITC, and the specific parameters are shown in Table 2. The activation function used for this model is trained, and the other parameters of the network are: learning rate h = 0.01, training number epochs = 1000, target error goal = 10^−6^, training step show = 2, and the training samples are 300 interfacial thermal conductivity and mixing defect data (vacancy, doping, temperature) of heterogeneous structures calculated from molecular dynamics simulations are used as the input data set, and the Log-Sigmoid function is used as the transfer function for training and prediction.

## 3. Results

### 3.1. Effect of Doped

**Figure 2:**
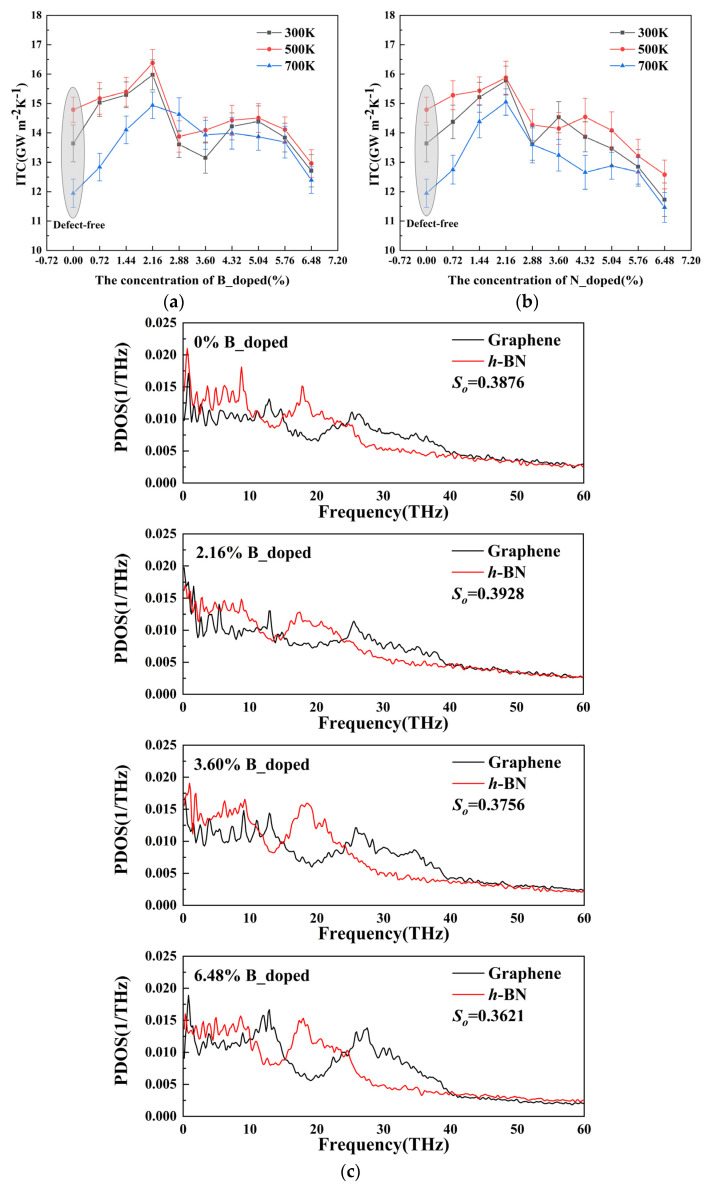

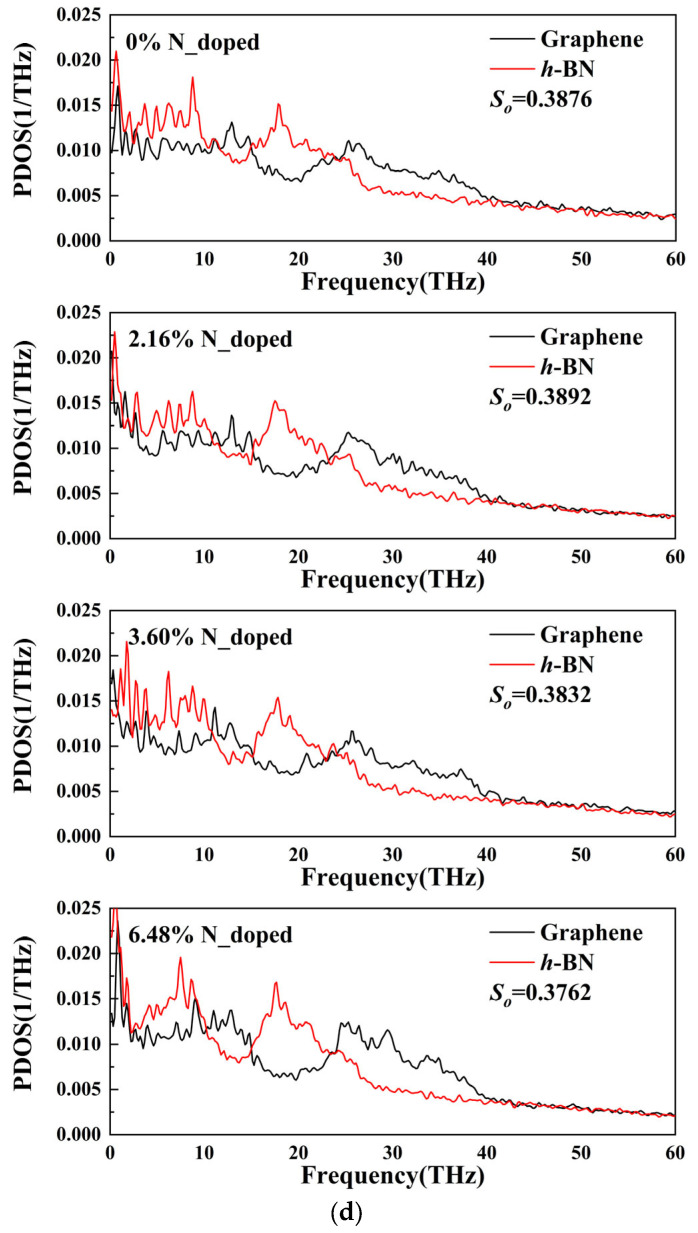
Effect of atomic doping on ITC. (**a**) Relationship between ITC and B-atom doping concentration. (**b**) Relationship between ITC and N-atom doping concentration. (**c**) PDOS plots at different B-atom doping concentrations. (**d**) PDOS plots at different N-atom doping concentrations.

In this subsection, we investigate the effect of the doping concentration of B and N atoms in the heterogeneous structure on the ITC of Gr/*h*-BN at different temperatures. Here, the ratio of doped atoms to available atoms in other regions of the heterojunction is used as a measure of the atomic doping concentration. As can be seen in Figure 2a, the ITC of Gr/*h*-BN gradually enhances and then shows a decreasing trend as the B-atom doping concentration increases and reaches a peak at 2.16% B-atom doping concentration. For example, at a temperature of 300 K, 2.16% B-atom doping can increase the ITC to 15.7 Gw m^−2^K^−1^. Figure 2b shows that the trend of ITC for the N atom doping case also follows the same trend of increasing and then decreasing, while the ITC at the heterogeneous interface reaches a maximum value of 15.98 Gw m^−2^K^−1^ when the N atom doping concentration is 2.16%. It is noteworthy that the variation in ITC follows the above trend regardless of the ambient temperature and the ITC increases with increasing temperature. This is because more high-frequency phonons are excited as the temperature rises, adding more carriers for interfacial thermal transport. Interestingly, as the temperature rises, the anharmonicity of atomic interactions at the contact likewise does as well. As a result, inelastic phonon scattering increases the phonon transmission coefficient. The ITC shows a decreasing trend when the temperature is increased to 700 K. This temperature effect has been confirmed by previous studies [33]. To elaborate the mechanism of ITC variation, we calculated the phonon density of states PDOS and the off-plane phonon coupling degree, so at the near interface (out-of-plane phonons dominate the interfacial heat transfer channel) we were able to discover the intrinsic mechanism of ITC variation for different B- and N-atom doping concentration conditions.

As shown in Figure 2c, the out-of-plane overlap factor *S_o_* rises and then gradually decreases with the increase in B-atom doping concentration, for example, *S_o_* is 3.928 and 3.621 when the B-atom doping concentration is 2.16% and 6.48%, respectively. This is consistent with the above ITC variation trend, which indicates that the increase in ITC is due to the enhanced interfacial phonon coupling by B-atom doping. Further, we found that in the mid-frequency region (15–25 THz), the PDOS of graphene shows a trend of increasing and then decreasing with the increase in B-atom doping concentration, which reveals that the thermal control of the Gr/*h*-BN heterogeneous interface can be achieved by changing the phonon density of states in the mid-frequency region (15–25 THz). It is noteworthy that the decrease in *S_o_* is larger for N-doping compared to B-doping, mainly due to the larger relative molecular mass of N atoms, which in turn has a more dramatic effect on the thermal conductivity of the heterogeneous interface, and the effect is more drastic.

### 3.2. Effect of the Single Vacancy

**Figure 3:**
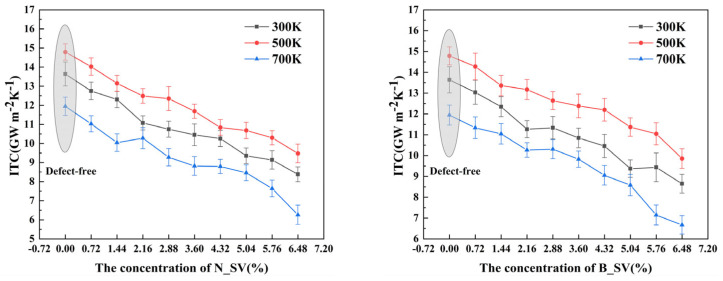

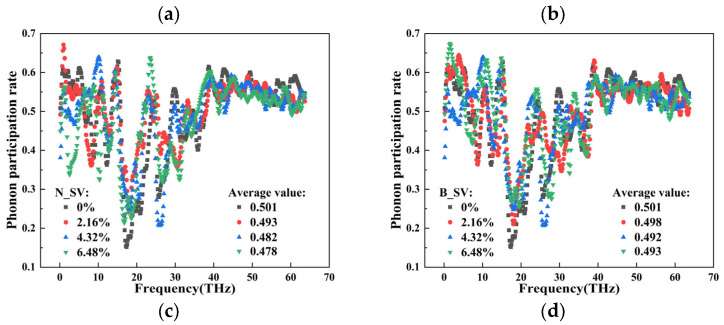
Effect of atomic single vacancies on ITC. (**a**) Relationship between ITC and N atomic single vacancy. (**b**) Relationship between ITC and B atomic single vacancy. (**c**) PPR at different N atomic vacancy concentrations. (**d**) PPR at different B atomic vacancy concentrations.

This section discusses the effect of single vacancies of B and N atoms in heterojunctions on ITC. For comparison with the above calculations, the system dimensions were kept constant (200 Å × 50 Å) and the temperatures were set to 300–700 K. The ITC values based on the concentrations of the two types (i.e., N_SV and B_SV) in the heterojunction are shown in Figure 3a,b. It can be seen that the ITC decreases linearly as the concentration of single vacancy defects increases, this has the same trend as the previously reported (from ∼(1.8 ± 0.2) × 10^3^ W mK^−1^ to ∼(4.0 ± 0.2) × 10^2^ W mK^−1^ near room temperature) intrinsic thermal conductivity of graphene with defective states [34], and the method of measuring the thermal conductivity of thin films based on Raman spectroscopy proves the correctness of this trend [35]. The ITC of SV-N and SV-B decreased from 13.6385 Gw m^−2^K^−1^ to 8.377 Gw m^−2^K^−1^ and 8.646 Gw m^−2^K^−1^, respectively, when the single vacancy defect concentration increased from 0 to 6.48%. According to the research, this is mostly because of increased phonon scattering at the interface brought on by vacancy defects as well as decreased phonon transmittance, while at the same time, the atomic vacancies introduce dangling bonds around the defects, completely destroying the integrity of the heterojunction plane and possibly leading to a reduction in active sites, which negatively affects the ITC of the heterogeneous structure. The linear relationship between ITC and vacancies indicates that covalent bonds, i.e., C-B bonds at the interface, are essential to the heat transport channel. According to the results, it can be found that the ITC values of SV-B are higher than those of SV-N as the concentration of single vacancies increases, regardless of the ambient temperature, for example, 9.855 Gw m^−2^K^−1^ and 9.476 Gw m^−2^K^−1^ for a vacancy concentration of 6.48% at a temperature of 500 K. This indicates that ITC is more sensitive to the introduction of N atomic vacancies than B atomic vacancies, and therefore we can regulate the heat conduction by introducing N atom vacancies at the interface.

To further comprehend how a single vacancy affects ITC and the distinction between N_SV and B_SV, we calculated the PPR in the near-interface region in Gr/*h*-BN at a temperature of 300 K and observed a significant difference between them. As shown in Figure 3c,d, the overall PPR decreases with increasing vacancy defect concentration as the single vacancy is introduced in the heterojunction, and the PPR of B_SV defects is higher than that of the heterogeneous structure with SV-N defects, which is consistent with the above ITC observations. For example, when the single vacancy concentration increases from 0 to 6.48%, the average PPR of N_SV and B_SV decreases to 0.478 and 0.493, respectively. Additionally, in the low-frequency region of 0–13 THz, as the single vacancy defect concentration increases, more and more phonons have a PPR below 0.4, whereas theory suggests that a PPR below 0.4 leads to a localized characteristic of phonons and hinders phonon thermal transport. Although the PPR of phonons increases with the increase in single vacancy defect concentration in the mid-frequency region (15–20 THz), the competition between the two (low and mid frequencies) leads to a significant decrease in the average PPR, i.e., it shows a linear decrease in the ITC of the heterogeneous structure, which indicates that phonons in the low-frequency region dominate the interface thermal transport channel. Thus, the above analysis suggests that the increase in the concentration of atomic single vacancy defects in the Gr/*h*-BN heterogeneous structure leads to a more severe phonon mode localization feature, resulting in a blockage of phonon thermal transport at the interface, which in turn leads to a gradual decrease in ITC.

### 3.3. The Prediction of ITC

**Figure 4:**
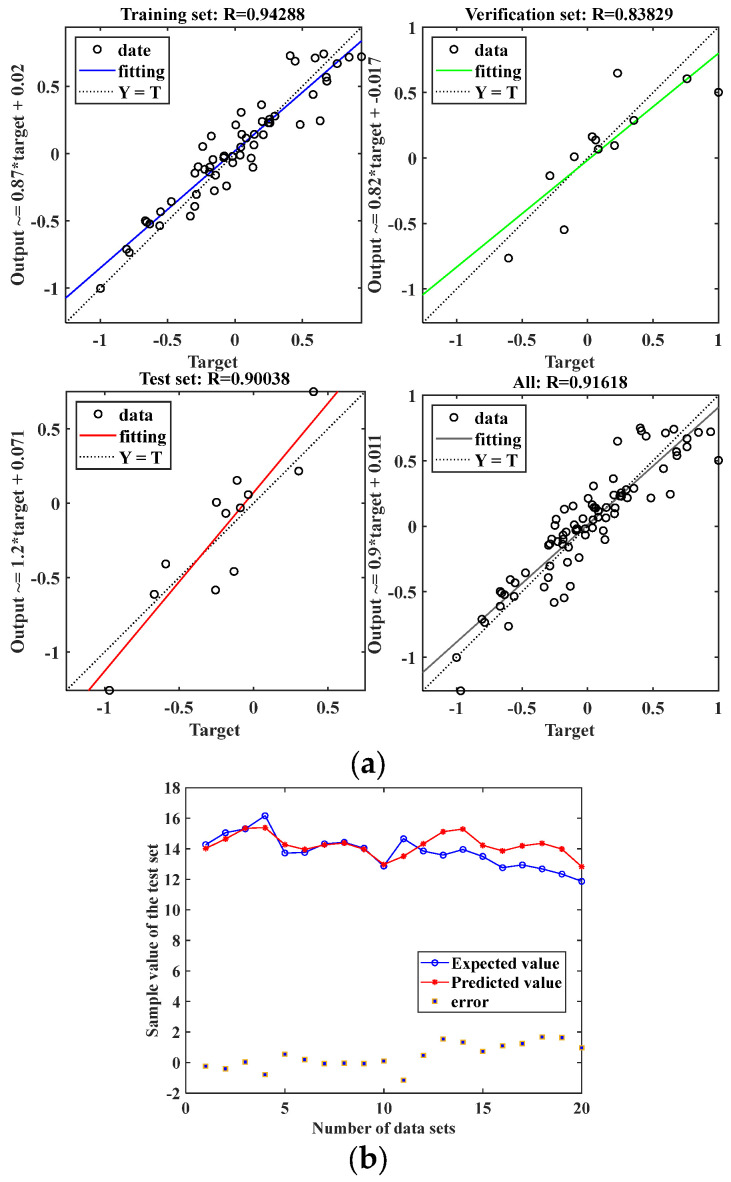
The results of BP neural network prediction. (**a**) *R*-value of each data set. (**b**) Fitting of predicted and real simulated calculated values.

Previous studies [36] have shown that molecular dynamics simulations are time-consuming and costly to calculate the interfacial thermal conductivity, therefore, in this paper, the interfacial thermal conductivity of Gr/*h*-BN heterogeneous structures under mixed defects is predicted by BP neural networks. To find the optimal number of hidden layers as well as the number of neurons, this is performed by comparing the average of Pearson correlation coefficients (*R*) between the simulated calculated and predicted values of the training and prediction networks, where R values include the average of *R* values of the training set, validation set, test set and all data sets. A combined comparison of the *R*-values of the training and prediction networks reveals that the results of the training network are slightly better when the number of implied layers is three as opposed to one or two. From Figure 4c, it can be seen that the R-values of both training and prediction networks are greater than 0.9 when we use the network specifications (5, 20, 20, 20, 1), where the *R*-value of all data sets is 0.91618, which can be used in the ITC prediction model. The *R* is calculated by (6).

(6) $$R=\frac{\sum_{i=1}^{n}\left(S_{1}-\bar{S_{1}}\right)\left(S_{2}-\bar{S_{2}}\right)}{\sqrt{\sum_{i=1}^{n}{\left(S_{1}-\bar{S_{1}}\right)}^{2}\sum_{i=1}^{n}{\left(S_{2}-\bar{S_{2}}\right)}^{2}}}$$

where *S*_1_ is the ITC value calculated by molecular dynamics simulation, *S*_2_ is the predicted value, $\bar{S_{1}}$ and $\bar{S_{2}}$ is the average of the ITC of the actual and predicted values, respectively, and *n* is the number of training samples of the model.

In this paper, we use Mean Square Error (*MSE*) to quantitatively characterize the accuracy of the training model, and Equation (7) is its mathematical expression.

(7) $$MSE=\sum_{i=1}^{n_{t}}\frac{{\left(y_{t}^{i}-y_{p}^{i}\right)}^{2}}{n_{t}}$$

where $y_{t}^{i}$ denotes the actual value, $y_{p}^{i}$ denotes the predicted value, and $n_{t}$ denotes the amount of data.

After several training sessions using the above network specifications, when the number of iterations is 120, the *MSE* at this time is 4.1696 × 10^−6^, which reaches the target error, and the generalization ability of the convolutional model is the best at this time, indicating that the training results of this model have met our needs for prediction accuracy, and the increase in the number of training sessions may lead to overtraining and overfitting phenomenon, and the decrease in the number of iterations of calculation may result in the under-fitting. Using ITC as the target of model validation and error analysis, the comparison between the predicted values and the actual simulated calculated values of the prediction model is illustrated in Figure 4b, it is clear from the picture that the projected values are consistent with the changing trend of the simulated calculated values, which better reflects the change characteristics of ITC under mixed defects. Overall, the prediction errors are small, all below 2%. The above error analysis results prove the reasonableness of the input factors selected and the prediction model constructed in this paper and verify that the model established in this paper is effective in predicting the ITC of mixed defect state heterojunctions and can be used for the prediction of the ITC of the Gr/*h*-BN interface. Using machine learning to predict the ITC of heterogeneous structures avoids the complex numerical solution process in molecular dynamics simulations and learns the material characteristic parameters in depth with the help of neural networks, thus realizing fast prediction of the thermal conductivity of heterogeneous interfaces, and this method will provide guidance and reference for the design and optimization of ITC of more complex defect-state heterogeneous structures.

## 4. Conclusions

In conclusion, the interfacial thermal energy transport of GR/*h*-BN heterostructures can be modulated by doping and vacancies of N and B atoms. The doping enhancement mechanism and vacancy regulation mechanism of GR/*h*-BN heterostructures were systematically investigated by using the NEMD method. The analysis of the results shows that the ITC of the heterogeneous structure can be appropriately enhanced by atomic doping, and the ITC increases and then decreases with the increase in the doping concentration of N- and B-atoms, and when the doping concentration reaches 2.16%, the ITC is the maximum value of 15.98 Gw m^−2^K^−1^ and 15.7 Gw m^−2^K^−1^, respectively, and the doping mainly enhances the ITC by increasing the phonon mode overlap in the mid-frequency region (15–25 THz). Atomic single vacancies can significantly reduce the ITC of Gr/*h*-BN heterojunctions, for example, when the vacancy concentration increases from 0 to 6.48% at a temperature of 500 K, the ITC values for N and B atomic vacancies are 9.476 Gw m^−2^K^−1^ and 9.855 Gw m^−2^K^−1^, respectively, which is because that in the low-frequency region (0–13 THz), as the concentration of single vacancy defects increases, more and more phonons have PPR below 0.4, leading to the localized characteristics of phonons and hindering phonon thermal transport. Meanwhile, we successfully constructed a BP neural network model that can rapidly predict the ITC of two-dimensional graphene/boron carbide heterostructures, and the average values of Pearson correlation coefficient (*R*) for the training and test sets reached 0.94 and 0.90, respectively, and the prediction error of the model was less than 2%.

## Figures and Tables

**Table 1:** The optimized Tersoff interatomic potential parameters for C, N, and B [29,30].

Parameters	C	N	B
*A* (eV)	1393.6	128.86866	40.0520156
*B* (eV)	430	138.77866	43.132016
λ (Å^−1^)	3.4879	2.8293093	2.2372578
μ (Å^−1^)	2.2119	2.6272721	2.0774982
β	0.00000015724	0.019251	0.0000016
*n*	0.72751	0.6184432	3.9929061
*c*	38.049	17.7959	0.52629
*d*	4.3484	5.9484	0.001587
*h*	−0.930	0.0000	0.5000
*R* (Å^−1^)	1.95	2.0	2.0
*D* (Å^−1^)	0.15	0.1	0.1

**Table 2:** Neural network model parameters.

Parameters		Range of Values	Unit
Input Factor	N_SV	0–6.48	%
	B_SV	0–6.48	%
	N_doped	0–6.48	%
	B_doped	0–6.48	%
	Temperature	300–700	K
Output Factor	ITC	6.73–14.97	Gw m^−2^K^−1^

## Data Availability

The raw/processed data required to reproduce these findings cannot be shared at this time as the data also forms part of an ongoing study.
